# Low T2-star (T2*) Signals in cardiac MR imaging in patients with dilated cardiomyopathy and sarcoidosis

**DOI:** 10.1186/1532-429X-17-S1-Q73

**Published:** 2015-02-03

**Authors:** Yumiko Kanzaki, Masako Yuki, Nobukazu Ishizaka

**Affiliations:** 1 Cardiology, Osaka Medical College, Osaka, Japan; 2 Radiology, Osaka medical college, Osaka, Japan

## Background

T2 star (T2*)-weighted MR imaging can be utilized to semi-quantitatively analyze tissue iron content. Excessive cardiac iron may aggravate cardiac performance, however, little is known about the T2* signal intensity in myopathic heart. We analyzed T2* signal intensity in primary and secondary cardiomyopathy.

## Methods

We enrolled 11 non-cardiomyopathic and 93 cardiomyopathic patients with following diagnoses: hypertensive or hypertrophic cardiomyopathy (n=22), dilated cardiomyopathy (n=28), sarcoidosis (n=13), old myocardial infarction (n=7), and other form of cardiomyopathy (n=23). Using 3-Teslar cardiac MRI (SignaHDxt, GE medical), T2* signal intensity in the cardiac ventricular septum and hepatic parenchyma were measured.

## Results

Compared with the cardiac T2* values in non-cardiomyopathic group (25.7±5.7 msec), those in hypertrophic cardiomyopathy or hypertensive cardiomyopathy (24.6±7.9 msec) did not differ significantly, however, those in the dilated cardiomyopathy (20.6±5.6 msec, P < 0.01) and sarcoidosis (18.0±3.2 msec, P < 0.01) were significantly lower. On the other hand, liver T2* values, as well as serum ferritin and iron concentrations, in patients with dilated cardiomyopathy or those with sarcoidosis did not differ those in the non-cardiomyopathic group.

## Conclusions

Cardiac T2* signal intensity was lower in the heart of dilated cardiomyopathy and sarcoidosis. Whether excessive iron in these forms of cardiomyopathy aggravates cardiac performance through generating noxious reactive oxygen species needs further investigation.

## Funding

N/A.

